# Loss of Nogo-A, encoded by the schizophrenia risk gene Rtn4, reduces mGlu3 expression and causes hyperexcitability in hippocampal CA3 circuits

**DOI:** 10.1371/journal.pone.0200896

**Published:** 2018-07-24

**Authors:** Stewart Berry, Oliver Weinmann, Ann-Kristina Fritz, Ruslan Rust, David Wolfer, Martin E. Schwab, Urs Gerber, Jeanne Ster

**Affiliations:** 1 Brain Research Institute, University of Zurich, Zurich, Switzerland; 2 Institute of Anatomy, University of Zurich, Zurich, Switzerland; 3 Department of Health Sciences and Technology, ETH Zurich, Zurich, Switzerland; Nathan S Kline Institute, UNITED STATES

## Abstract

Recent investigations of Nogo-A, a well characterized protein inhibitor of neurite outgrowth in the brain, have revealed additional functions including a role in neuropsychiatric disorders such as schizophrenia. Here we examined Nogo-A functions in mouse CA3 hippocampal circuitry. Patch clamp recordings showed that the absence of Nogo-A results in a hyperactive network. In addition, mGlu3 metabotropic glutamate receptors, which exhibit mutations in certain forms of schizophrenia, were downregulated specifically in the CA3 area. Furthermore, Nogo-A^-/-^ mice showed disordered theta oscillations with decreased incidence and frequency, similar to those observed in mGlu3^-/-^ mice. As disruptions in theta rhythmicity are associated with impaired spatial navigation, we tested mice using modified Morris water maze tasks. Mice lacking Nogo-A exhibited altered search strategies, displaying greater dependence on global as opposed to local reference frames. This link between Nogo-A and mGlu3 receptors may provide new insights into mechanisms underlying schizophrenia.

## Introduction

Nogo-A is a membrane-bound protein widely expressed in the central nervous system, most frequently in oligodendrocytes, but also in certain neuronal populations. Its distribution decreases strongly during early postnatal development except in highly plastic brain regions such as the hippocampus [1,2]. Blocking Nogo-A function in the hippocampus enhances LTP at Schaffer collateral synapses [3] and significantly alters CA3 dendritic architecture, both in complexity and length, predominantly in the region of recurrent collateral synapses [4]. Similar changes have been seen in cerebellar Purkinje cells [5] and layer 2/3 M1 cortical neurons [6]. In addition, the Nogo-A receptor NgR1 is important for regulating hippocampal neurite growth [7] and plasticity in the visual cortex [8].

Nogo-A, which is coded by the gene *Rtn4*, has been implicated in certain psychiatric illnesses. It is localized on chromosome 2p16, a hotspot for schizophrenic candidate genes [9,10] and Rtn4 polymorphisms have been detected in schizophrenia patients[11,12]. Furthermore, 22q11, an additional region associated with risk of schizophrenia, contains the coding region for NgR1[13,14]. Behavioral studies in rodents have revealed that Nogo-A^-/-^ mice and Nogo-A knock-down rats exhibit schizophrenic endophenotypes, such as decreased prepulse inhibition, impaired latent inhibition, and perseverative behavior [15, 16]. While theories have been put forth regarding Nogo-A's role in such behaviors, the underlying mechanisms remain unknown [17]. The observed changes in dendritic branching of CA3 pyramidal cells, however, may provide clues. The hippocampal alterations in the absence of Nogo-A are likely to affect cognitive functions relying on neuronal CA3 circuitry. We therefore examined the roles of Nogo-A in maintaining functional integrity in the CA3 hippocampal network. In its absence, CA3 pyramidal cells displayed an elevated excitation/inhibition ratio and disrupted theta rhythmicity associated with mGlu3 receptor downregulation, and Nogo-A^-/-^ mice displayed altered spatial navigation strategies, with greater dependence on global reference frames.

## Materials and methods

### Slices cultures

Organotypic slices were prepared from 6-day-old Nogo-A^-/-^ and wild-type (WT) mice as previously described [18], following a protocol approved by the Veterinary Department of the Canton of Zurich of Animal Care (approval ID 81–2014). All mice used in this study (wild type and transgenic) were of the C57BL/6 background. Transgenic lines were maintained as homozygous. Animals were kept in climate controlled rooms with enriched housing and given ad libitum access to food and water. Both sexes of mice were used in a randomized fashion resulting in an approximately 50/50 ratio. Approximately three mice were used for each slice culture preparations and each experimental condition was tested over the course of at least three preparations. With slices chosen at random, we estimate that electrophysiological data is derived from a minimum of five different animals per genotype. Hippocampal slice cultures were maintained in a roller drum incubator at 37°C for a minimum of three weeks before recording. For some experiments, cultures were prepared according to the Stoppini method [19]. No differences in experimental data were detected between slices prepared by either culturing method.

### Electrophysiology

After 3 weeks *in vitro*, slice cultures were transferred to a recording chamber on an upright microscope (Axioscope FS; Zeiss, Oberkochen, Germany). Slices were superfused continuously at 1ml min^-1^ with ACSF containing (in mM) 125 NaCl, 2.7 KCl, 11.6 NaHCO_3_, 0.4 NaH_2_PO_4_, 1 MgCl_2_, 2 CaCl_2_, 5.6 D-glucose, and 0.001% phenol red (pH 7.4, osmolarity 305 mOsm) at 33°C. Whole-cell recordings were obtained from cells held at -70 mV with an Axopatch 200B amplifier (Axon Instruments, Union City, CA, USA). Recording pipettes (4–6 MΩ) were filled with (in mM) 120 potassium gluconate, 5 KCl, 10 Hepes, 1 EGTA, 5 phosphocreatine, 0.07 CaCl_2_, 2 Mg-ATP, 0.4 Na-GTP (pH 7.2, osmolarity 290–310 mOsm.). Membrane potentials were corrected for junction potentials.

To determine AMPAR/NMDAR ratios, cells were held at −70 mV for 10 sweeps to record electrically evoked fast AMPA receptor-mediated currents (EPSCs). Subsequently, cells were held at +30 mV, where synaptic currents consist of a slow NMDA receptor-mediated current that follows the fast AMPA receptor component. To assess the NMDA component, current amplitude were measured 70 ms following the extracellular stimulation.

Spontaneous activity, mGlu3 receptor-mediated currents, and theta oscillations were filtered at 2–5 kHz and analyzed off-line (pCLAMP 10; Axon Instruments). Oscillation analyses were performed after 6 min of application of LCCG-I or MCh. Calculation of oscillatory activity was performed from the time of the second peak in the Clampfit autocorrelation function. A segment was considered rhythmic when the second peak of the autocorrelation function was at least 0.3 and several regularly spaced peaks appeared.

### Drugs

DCG-IV, LCCG-1 and LY341495 were purchased from Tocris Bioscience (Bristol, United Kingdom). TTX, NBQX, D-AP5 and methacholine chloride were purchased from Abcam (Cambridge, United Kingdom).

### Densitometric analysis

40 μm coronal sections were stained overnight in primary antibody mGlu2/3 (Rabbit 1:250; Merck Millipore) diluted in the blocking TNB-Tris Buffer and 0.05% Triton at 4°C. After washing in PBS and Cy3-conjugated secondary antibody incubation for 1h, we mounted the PBS-washed section on slides in 50mM Tris pH 8.0, let the sections dry, and coverslipped with Mowiol. Images were acquired using a cooled CCD Camera (Coolsnap HQ, Photometrics, Tucson, Arizona, USA) attached to an Axiophot microscope (Zeiss) and collected using image analysis software (MCID Elite Software 7.0, Imaging Research, St Catharines, Canada). The immunoreactivity in a given region was obtained from densitometric measurement of the mean gray value using ImageJ (https://imagej.nih.gov/ij/). The background corrected optical densities were averaged per hippocampus region and animal. For verification of mGlu2/3 antibody specificity, the same staining procedure was followed, but an overnight incubation of the antibody with a synthetic mGlu2/3 fragment (Merk, Millipore) (50ug/mL) was performed at 4°C before applying to hippocampal tissue.

### Immunofluorescence localization of Nogo-A

Mice (approximately 3-weeks-old) were anesthetized with Nembutal and fixed by perfusion through the heart with 4% buffered PFA. Brains were postfixed overnight at 4°C, cryoprotected in 30% sucrose, and sectioned at 40 μm in a cryostat. For all staining, free-floating coronal sections were used. For subcellular localization of Nogo-A in the CA3 region, immunofluorescence double staining with the dendritic marker MAP-2 was performed. Sections were incubated overnight at 4°C with primary antibodies against Nogo-A (Rabbit Serum “Laura”1:250) and MAP-2 (Mouse 1:250; Merck Millipore, Darmstadt, Germany) diluted in TNB-Tris Buffer and 0.05% Triton. After 3 washes for 10 minutes at room temperature, secondary antibodies coupled to indocarbocyanine Cy3 (1:200 Jackson Immunoresearch Laboratories, West Grove, PA, USA) or to Alexa Fluor 488 (Life Technologies, Paisley, United Kingdom) were applied. After final washing steps in PB, sections were mounted and coverslipped with Mowiol (Merck Millipore). For NeuN and Nogo-A colocalization assays, secondary antibodies were conjugated to Alexa-488 (NeuN) and Cy3 (Nogo-A). Epifluorescence was analyzed in a sequential image acquisition mode (TCS-SP2; Leica, Heerbrugg, Switzerland) using 10×, 20×, 40× and 63× objectives.

### Evaluation of dendritic morphology

CA3 pyramidal cells from organotypic slice cultures (21–28 DIV) were patch clamped in whole-cell mode to allow dialysis of 0.1–0.2% biocytin for 20 minutes. Slices were then fixed overnight in 4% PFA at 4°C followed by three 30 minute washes in PBS at room temperature. Blocking and permeabilization was achieved by incubating in 10% horse serum, 0.05% triton x-100, 1x PBS pH 7.4 for 12–24 hrs at 4°C. Slices were then stained with a streptavidin-conjugated Alexa-546 fluorophore (Life Technologies) diluted 1:1000 in 5% horse serum, 0.05% triton x-100, 1x PBS pH 7.4 for 12–15 hrs at 4°C. Three 30 minute washes in PBS were then performed at room temperature and slices were mounted with Dako fluorescence mounting medium (Dako, Glostrup, Denmark). Cells were imaged using a TCS-SP2 confocal microscope (Leica, Heerbrugg, Switzerland). Apical dendrites were reconstructed and analyzed with Sholl analysis using ImageJ.

### Electron microscopy

We used pre-embedding immunolocalization techniques. To detect Nogo-A we used the DAB-Ag-Au substitution method as described elsewhere [5]. Sections were osmicated and, after uranyl acetate contrast incubation, embedded in epoxy. Ultrathin sections of 100 nm were imaged with a Zeiss EM10 electron microscope. Analysis of Nogo-A distribution was performed as previously described [6]. Briefly, 20 micrographs in the CA3 *stratum radiatum* region from each of two animals were chosen at random and imaged at 63,000x. The volume densities of five compartments were determined stereologically by overlaying a point grid matrix (4 x 4) to count and calculate the Relative Labeling Index of DAB-Gold immunoreactive positive compartments. Compartments were determined to be either dendrites, spines, axons, presynaptic terminals, or other (glia, artifact, etc.). Quantification of labeling was performed as described elsewhere [20].

### Morris water maze

Maze 1 utilized a white circular pool (150 cm in diameter, 50 cm tall) with water made opaque by the addition of milk and maintained at 24 ± 1°C. Four black paper cues of distinct shape were placed on the room walls. 19-22-week-old mice were randomly separated into four cohorts and trained to find a grated, square platform (16 x 16 cm) located 1 cm below the water surface in one of four radial quadrants. 13 WT (9 female, 4 male) and 11 Nogo-A^-/-^ (6 female, 5 male) animals were used. Animals were kept in climate controlled rooms with enriched housing and given ad libitum access to food and water. Training entailed six trials per day separated by 40 minutes during their dark cycle. Mice were released from pseudorandom positions and given up to 2 minutes to locate the platform. Probe trials were performed in the mornings before training, during which animals were allowed to search one minute for the phantom platform. Release points were 225° CW from the goal position. Full-cue probe trials were performed on days 11 and 12. Single and no-cue probes were performed on days 13 and 14, respectively. For single-cue probes, the remaining cue was positioned 135° CW from the phantom platform.

Maze 2 used the same pool as maze 1; however, cues and lighting conditions were adjusted to increase the saliency of distal cues, while limiting local and extraneous distal information. The pool was enclosed by black curtains displaying three-dimensional cues of distinct shape and color. Four floor lights focused onto the center of cues with intensities of 25–50 lux. A small camera and LED light were mounted to the ceiling of the maze. The light was centered above the pool and provided evenly-distributed low-level illumination to accommodate tracking software (~8 lux on water surface). To reduce stress, mice were pre-handled for one week and experiments were carried out by the same individual. 10 WT (6 female, 4 male) and 13 Nogo-A^-/-^ (6 female, 7 male) mice were used. Training and probe trials were conducted as in maze 1. Full-cue probes were performed on days 14–15, single-cue on days 16–17, no-cue on days 18–19 and the final full-cue condition on days 20–21. As in maze 1, probe trials were always followed by 6 training sessions to reduce extinction.

Tracking was carried out at 4.2Hz with a 576 x 768 pixel resolution camera using a Noldus EthoVision 3.0 system (Noldus Information Technology, Wageningen, The Netherlands) and data analyzed offline (Wintrack, http://www.dpwolfer.ch/wintrack). The first 30 seconds of probe trials were analyzed, as mice tended to adopt chaining strategies in the latter half, a behavior observed by others [21].

### CA3 tissue dissection and real-time quantitative PCR

Mice aged 18–27 days were anesthetized with isoflurane and sacrificed by decapitation. Brains were rapidly removed and CA3 was dissociated from the hippocampus on ice. Tissue was flash-frozen in liquid nitrogen and stored at -80°C until processing. RNA extraction was performed using the RNeasy mini kit (Qiagen) according to the manufacture’s recommendations. cDNA libraries were generated using the TaqMan reverse transcription kit (Roche) as per the manufacturer’s instructions and stored at -20°C until processing. RT-qPCR was performed using SYBR Green I (Roche) with a Light Cycler 480 II (Roche) and normalized to *Gapdh*. Specificity of the dissection was performed by comparing expression of the CA3 marker G*rik4* in presumed CA3 tissue to CA1/DG tissue. The following primers were used (in 5’ to 3’ direction): *Grm3* For: TGGAGTTTGTCAGAGCATCG Rev: GATTTGTCGCTGAGTTTGGC. *Gapdh* For: CAGCAATGCATCCTGCACC Rev: TGGACTGTGGTCATGAGCCC. *Grik4* For: CGGAACGTGAGAAAGTGATCG Rev: CTAGAAGCATGAAGAGCCAGAC.

### Statistical analyses

Statistical analyses were performed using IBM SPSS (IBM Corp., Armonk, NY, USA). Values are represented as mean ± SEM. Normalcy of distribution was determined via the Shapiro Wilk test. Except where noted, normally distributed data was analyzed via t-tests. For data sets with non-normal distributions, non-parametric tests were used (Mann-Whitney U test for independent samples or Wilcoxon signed-rank test for paired samples). Significance was defined as *P ≤ 0.05, **P ≤ 0.01, ***P ≤ 0.001.

## Results

### CA3 pyramidal cells in Nogo-A^-/-^ hippocampus exhibit increased spontaneous synaptic activity

**Fig 1:**
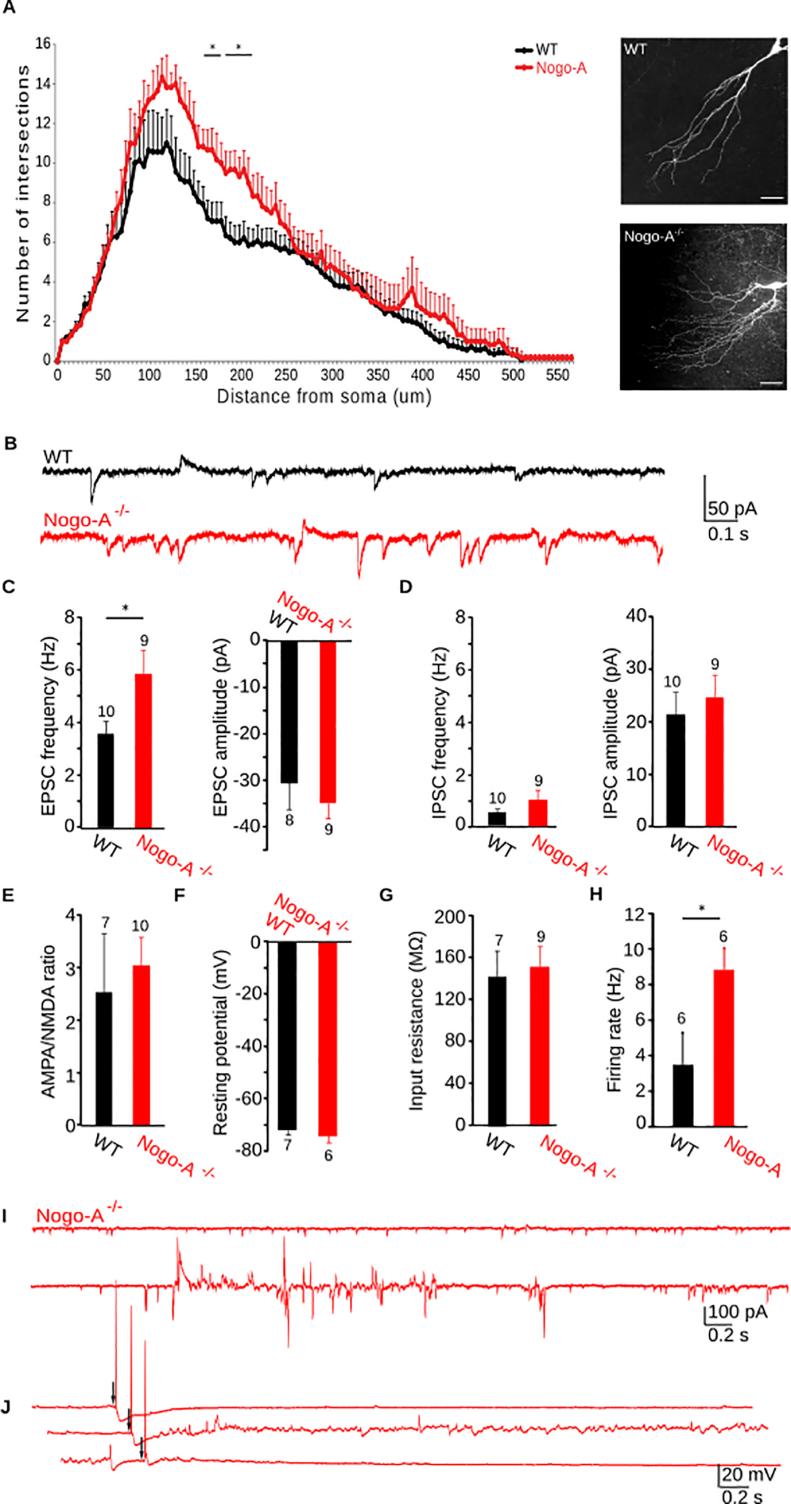
CA3 dendritic morphology and network excitation are enhanced in Nogo-A^-/-^ hippocampus. (A) Left, Apical dendritic arborization is increased in Nogo-A^-/-^ CA3 pyramidal cells. Images of CA3 pyramidal cell apical dendrites. (B) Representative activity recorded in a CA3 pyramidal cell voltage-clamped at -70 mV from WT and Nogo-A^-/-^ slice cultures. (C) EPSC frequency, but not amplitude, is increased in Nogo-A^-/-^ cells. (D) Neither IPSC frequency nor amplitude is significantly altered. (E) The AMPA/NMDA response ratio at CA3 recurrent collateral synapses was determined by stimulating fibers in stratum radiatum while recording from CA3 pyramidal cells voltage-clamped at -70 mV to measure AMPA currents and at +30 mV to measure NMDA currents. The AMPA/NMDA ratio does not differ between Nogo-A^-/-^ and WT CA3 recurrent collateral synapses. (F) Resting membrane potential and (G) input resistance are unaltered in CA3 pyramidal cells from Nogo-A^-/-^ hippocampus. (H) The firing rate in response to 550 pA current injections is significantly increased in Nogo-A^-/-^ pyramidal cells. (I)Activity in Nogo-A^-/-^ CA3 pyramidal cells is marked by periods of aberrant discharge. (J) Synaptic stimulation before (upper trace), during (middle trace), and after (lower trace) aberrant discharge in Nogo-A^-/-^ hippocampus does not provoke epileptiform activity. Bars indicate S.E.M.. Number of cells is indicated above bar graphs. Paired t-test for all, except D, where a Mann-Whitney was performed.

We hypothesized that the increased CA3 dendritic arborizations reported in Nogo-A^-/-^ mice [4] would allow for the formation of more synaptic inputs, which may enhance activity levels. We first confirmed the results of Zagrebelsky et al. by performing Sholl analyses of reconstructed apical dendrites in pyramidal cells (Fig 1A). CA3 cells from Nogo-A^-/-^ mice had significantly more dendritic arborizations between 165 and 210 um from the soma (*P* = 0.014 to 0.05), corresponding to the *stratum radiatum*, where recurrent collateral synapses occur.

We then examined EPSCs and IPSCs in CA3 pyramidal cells using 21–28 DIV organotypic hippocampal slices. Cultured slice preparations were chosen over acute slices to preserve network circuitry and to avoid injury-induced activation of Nogo-A. Amplitudes of EPSCs and IPCSs were similar in WT and Nogo-A^-/-^ slices (WT-EPSCs = -30.7 ± 5.7 pA, Nogo-A^-/—^EPSCs = -29.3 ± 9.1 pA, *P* = 0.516; WT-IPSCs = 21.5 ± 4.2 pA, Nogo-A^-/—^IPSCs = 24.7 ± 4.3 pA, *P* = 0.222; Fig 1B–1D). However, the spontaneous frequency of EPSCs, but not IPSCs, was increased in the slices from Nogo-A^-/-^ mice (WT-EPSCs = 3.6 ± 0.5 Hz, Nogo-A^-/—^EPSCs = 5.9 ± 1 Hz, *P* = 0.0383; Fig 1C; WT-IPSCs = 0.5 ± 0.2 Hz, Nogo-A^-/-^ IPSCs = 1 ± 0.4 Hz, *P* = 0.230; Fig 1D). The modification of excitatory transmission may be due to altered expression of postsynaptic ionotropic glutamate receptors. However, we observed no difference between AMPA/NMDA ratios at CA3 recurrent collateral synapses from WT and Nogo-A^-/-^ hippocampus (WT-AMPA/NMDA = 2.5 ± 1.1, Nogo-A^-/—^AMPA/NMDA = 3 ± 0.6, *P* = 0.597; Fig 1E). The enhanced activity recorded in CA3 pyramidal cells was not the result of a change in resting membrane potential (Fig 1F) or input resistance (WT = -72 ± 1.7 mV, Nogo-A^-/-^ = -74.1 ± 2.5 mV *P* = 0.443; WT = 142 ± 24. 8 MΩ, Nogo-A^-/-^ = 151.7 ± 19.6 MΩ, *P = 0*.622; Fig 1G). However, neurons lacking Nogo-A fired significantly more action potentials in response to depolarizing input (WT = 3.5 ± 1.78 Hz, Nogo-A^-/-^ = 8.83 ± 1.22 Hz, *P* = 0.033; Fig 1H)

These findings suggest that the increase in synaptic activity in the absence of Nogo is not related to a change in postsynaptic properties but rather is the result of a greater number of synapses onto the more complex apical dendritic tree of these CA3 pyramidal cells [4]. This increased dendritic branching and associated enhancement in activity might be expected to trigger epileptiform activity. We did in fact observe aberrant bouts of activity consisting of high frequency EPSC and IPSCs (Fig 1I) that were never observed in WT cells. These epochs occurred at a rate of 0.66 ± 0.13 per five minutes and lasted approximately 5.3 ± 1.53 s; n = 10. However, synaptic stimulation during these periods failed to elicit seizure-like responses (Fig 1J).

**Fig 2:**
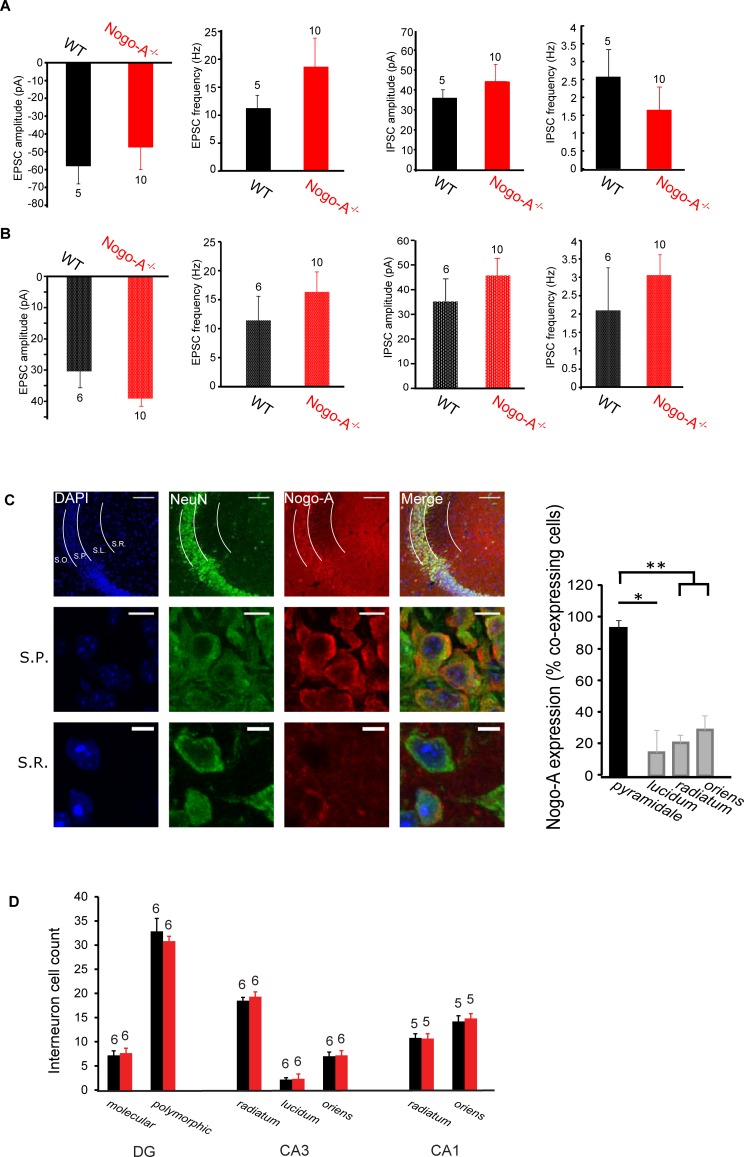
Nogo-A does not modulate CA3 interneuron expression or spontaneous activity and is predominantly expressed by pyramidal cells. (A) Neither EPSC nor IPSC frequency and amplitude are significantly altered in Nogo-A^-/-^ CA3 fast-spiking (>80 Hz) interneurons. Mann-Whitney test. (B) Same as A, but for slower-spiking interneurons (<80 Hz). Mann-Whitney test. (C) Immunohistochemistry staining reveals significantly more Nogo-A expression in neurons of the *stratum pyramidale* layer over those in other strata (*P* = 0.039 for *strata lucidum*, *P* < 0.01 for *stratas radiatum* and *oriens*). Scale bars represent 20 μm.T-tests. (D) Interneuron cell count in WT and Nogo-A^-/-^ hippocampi do not differ. T-tests.

We next compared the spontaneous activity of WT and Nogo-A^-/-^ CA3 interneurons. It has recently been reported that PV^+^ basket cells express Nogo-A[22]. To check for alterations of these fast-spiking interneurons, we analyzed activity from cells outside the *stratum pyramidale* with a firing frequency of >80 Hz in response to depolarizing current injections. No significant differences in event amplitude or frequency were detected in these putative PV^+^ interneurons (amplitude of EPSCs: WT = -57.14 ± 9.82 pA, Nogo-A^-/-^ = -46.81 ± 12.27, *P* = 0.14; frequency of EPSCs: WT = 11.19 ± 2.36 Hz, Nogo-A^-/-^ = 18.59, ± 5.19 Hz, *P* = 0.71; amplitude of IPSCs: WT = 36.04 ± 4.05 pA, Nogo-A^-/-^ = 44.28 ± 8.56 pA, *P* = 0.39; frequency of IPSCs: WT = 2.58 ± 0.76 Hz, Nogo-A^-/-^ = 1.65 ± 0.64 Hz, *P* = 0.18, Fig 2A). We also compared the activity among slower firing interneurons (< 80 Hz). As with fast-spiking cells, no significant differences were detected. (amplitude of EPSCs: WT = -30.39 ± 5.29 pA, Nogo-A^-/-^ = -39.12 ± 2.47 pA, *P* = 0.09; frequency of EPSCs: WT = 11.42 ± 4.15 Hz, Nogo-A^-/-^ = 16.33 ± 3.45 Hz, *P* = 0.20; amplitude of IPSCs: WT = 35.19 ± 9.21 pA, Nogo-A^-/-^ = 45.71 ± 7.04 pA, *P* = 0.25; frequency of IPSCs: WT = 2.10 ± 1.16 Hz, Nogo-A^-/-^ = 3.06 ± 0.56, *P* = 0.12, Fig 2B). Consistent with these data, an analysis of immunohistochemically stained sections revealed that Nogo-A in the CA3 area is largely restricted to cells in the *stratum pyramidale* (90.1%), the location of pyramidal cells and only few interneurons. In contrast, only 8.3% of neurons in the *lucidum*, 20.9% in the *radiatum*, and 28% in the *oriens* expressed Nogo-A (Fig 2C, S1 Table, S1 Fig). The few Nogo-A positive neurons in these strata may be parvalbumin interneurons, thought to express Nogo-A [22]. In addition, the total number of interneurons was unaltered in Nogo-A^-/-^ mice (Fig 2D and S1 Table).

We also examined the CA1 network for changes in spontaneous activity. However, we found no alterations in excitatory or inhibitory activity recorded in pyramidal cells (amplitude of EPSCs: WT = -39 ± 4.9 pA, Nogo-A^-/-^ = -35.5 ± 8.5 pA, *P* = 0.20; amplitude of IPSCs: WT = 43 ± 9.6 pA, Nogo-A^-/-^ = 32.1 ± 6.9 pA, *P* = 0.36; frequency of EPSCs: WT = 1.2 ± 0.2 Hz, Nogo-A^-/-^ = 1.1 ± 0.2 Hz, *P* = 0.93; frequency of IPSCs: WT = 0.2 ± 0.03 Hz, Nogo-A^-/-^ = 0.3 ± 0.1 Hz, *P* = 0.721; n = 6 for WT and n = 7 for Nogo-A^-/-^. This is in agreement with previous findings, where no changes in the dendrites of CA1 pyramidal cells were seen in the region of Schaffer collateral synapses [4].

### Synaptic localization of Nogo-A in the CA3 area

**Fig 3:**
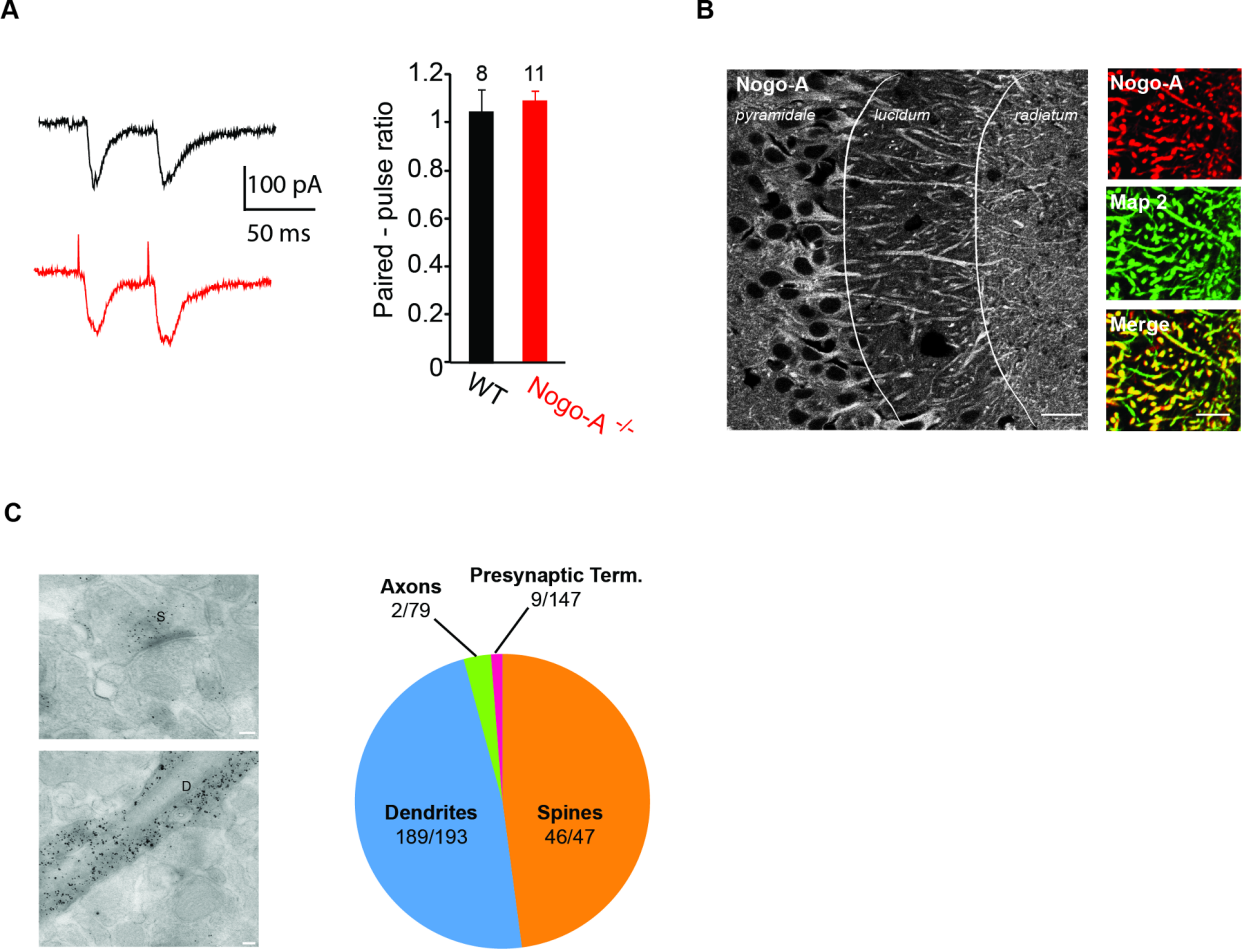
Nogo-A does not modulate presynaptic properties and is expressed in the postsynaptic neuronal compartment. (A) The synaptic paired-pulse ratio in response to CA3 recurrent collateral stimulation is not modified in recordings from CA3 pyramidal cells lacking Nogo-A, indicating unchanged probability of presynaptic glutamate release (t-test).(B) A hippocampal section through the CA3 region from a WT mouse stained for Nogo-A. Right, region of the *strata radiatum* showing colocalization with the dendritic marker Map2. Scale bars represent 20 μm. (C) Synaptic localization of Nogo-A in CA3 *stratum radiatum*. Left: An electron microscopic section reveals Nogo-A expression in a spine *(S)* and a dendrite *(D)* of a CA3 pyramidal cell, but not in presynaptic terminals. Scale bars represent 0.1 μm. Right: Proportion of structures expressing Nogo-A. The vast majority of Nogo-A staining is in spines and dendrites and not in axons or presynaptic terminals. Numbers refer to quantification of labeling for each compartment determined by imposing a 4x4 grid on each of 40 electron-micrographs (see Methods and Materials for procedure).

The observed increase in excitatory synaptic activity recorded in CA3 pyramidal cells from Nogo-A^-/-^ mice could involve a presynaptic mechanism. However, assessment of the paired-pulse ratio of EPSCs recorded in CA3 pyramidal neurons, as an indicator of synaptic release probability, revealed no difference between WT and Nogo-A^-/-^ cells (PPR at an interval of 50 ms: WT = 1 ± 0.1, Nogo-A^-/-^ = 1.1 ± 0.04, *P* = 0.641; Fig 3A). Accordingly, we observed Nogo-A expression only in postsynaptic compartments. Double labeling experiments showed colocalization of microtubule-associated protein 2 (MAP2), a dendritic marker, and Nogo-A (Fig 3B). This observation was confirmed by electron microscopy, which revealed the highest immunoreactivity for Nogo-A in spines and dendrites of CA3 pyramidal cells (Fig 3C and Table 1). No specific labeling was detected in presynaptic elements.

**Table 1:** In the *stratum radiatum* of CA3, Nogo-A is expressed postsynaptically.

	Immunoreactive compartments	Total compartments	Expected random labeling	RLI	χ2
Dendrites	189	193	74.2	**2.5**	177.7
Spines	46	47	18.1	**2.5**	43.2
Presynaptic terminals	9	147	56.5	0.2	39.9
Axons	2	79	30.4	0.1	26.5
Other (glia or artifact)	0	174	66.9	0	66.9

χ^2^ of 354.2 and 4 degrees of freedom, *P* < 0.001. Therefore, the distribution pattern differs significantly from random. The Relative Labeling Index reveals preferential expression within dendrites and spines (RLI > 1).

### Altered theta oscillations in the CA3 area of Nogo-A^-/-^ mice

**Fig 4:**
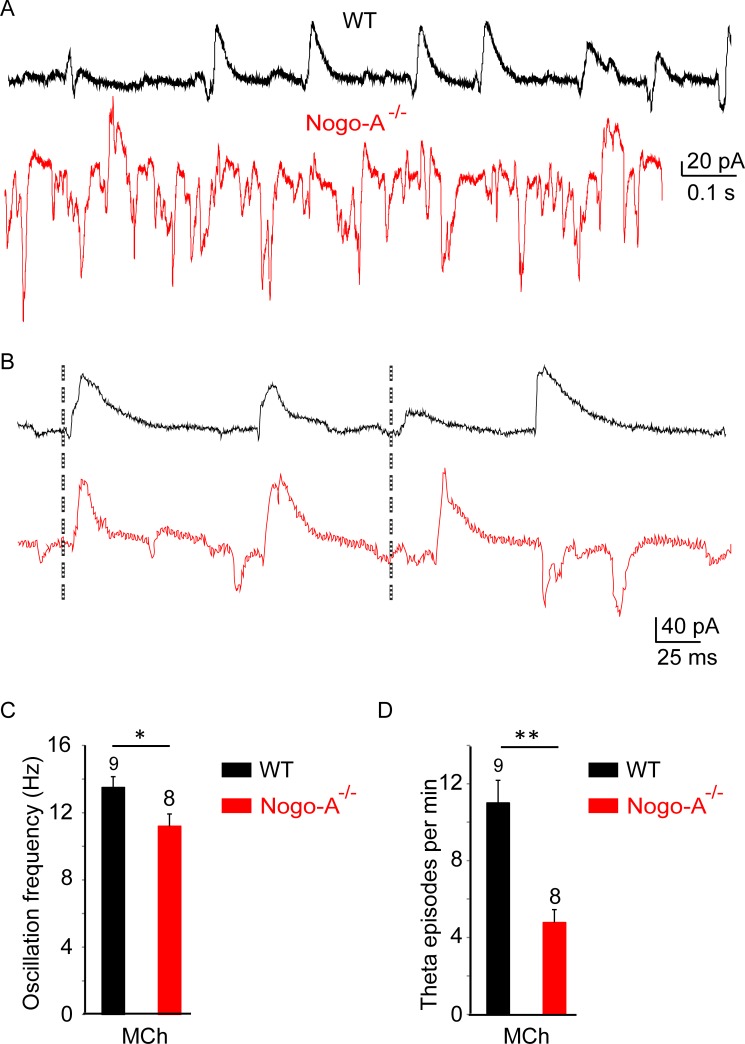
Methacholine-induced oscillations in the CA3 network are disrupted in Nogo-A^-/-^ hippocampus. (A) Representative traces of methacholine-induced oscillations recorded in CA3 pyramidal cells voltage-clamped at -70 mV from WT and Nogo-A^-/-^ hippocampus. Nogo-A^-/-^ slices often display erratic activity in response to methacholine. (B-C) When theta oscillations do occur in Nogo-A^-/-^ slices, their frequency is significantly reduced (Mann-Whitney test) (D). The incidence of theta episodes is much lower in Nogo-A^-/-^ recordings than in WT recordings (t-test).

The increase in the synaptic excitation/inhibition (E/I) ratio that we observed in our recordings in Nogo-A^-/-^ mice is likely to affect network properties, such as oscillatory activity. To determine whether increased neuronal excitability affects hippocampal CA3 network rhythmicity, we recorded from patch-clamped CA3 pyramidal cells in slice cultures where theta oscillations were induced by applying the muscarinic agonist methacholine (MCh, 500 nM, 20 min [23]). Cholinergic activation of CA3 networks in WT hippocampus results in cycles of rhythmicity [24,25]. Interestingly, the frequency of theta oscillations and of theta episodes was significantly reduced in Nogo-A^-/-^ mice compared to WT mice (frequency: WT = 13.8 ± 1.2 Hz, Nogo-A^-/-^ = 11.2 ± 0.7 Hz, *P* = 0.017; episodes/min: WT = 10.6 ± 1.7, Nogo-A^-/-^ = 5.25 ± 0.8, *P* = 0.005; Fig 4B–4D).

### Absence of Nogo-A downregulates the functional expression of metabotropic glutamate receptor 3 (mGlu3)

**Fig 5:**
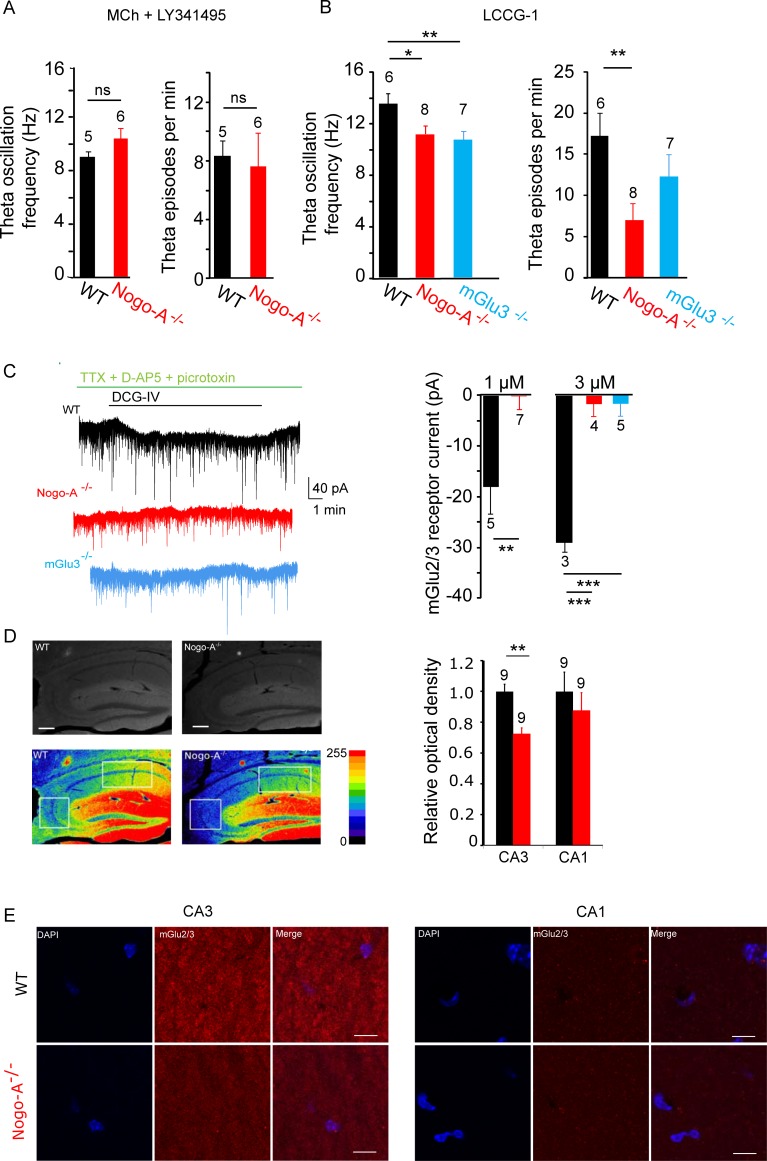
mGlu3 receptors are significantly downregulated in the CA3 area of Nogo-A^-/-^ mice. (A) The lower frequency of methacholine-induced theta oscillations in the Nogo-A^-/-^ CA3 network, as compared to WT (Fig 4C), is no longer observed in the presence of the mGlu2/3 receptor antagonist LY-341495 (3 μM). Furthermore, LY-341495 has no significant effect on the incidence of methacholine-induced oscillations in Nogo-A^-/-^ hippocampus (ns = not significant). (B) Oscillatory activity induced by the mGlu2/3 receptor agonist LCCG-1 (10 μM) is significantly lower in Nogo-A^-/-^ hippocampus, with levels similar to those recorded in mGlu3^-/-^ cells. (C) Bath application of the mGlu2/3 receptor agonist DCG-IV in presence of TTX (1 μM), picrotoxin (100 μM), and D-AP5 (40 μM) induces inward current in voltage-clamped CA3 pyramidal cells. DCG-IV does not induce significant current in CA3 pyramidal cells from Nogo-A^-/-^ or mGlu3^-/-^ mice. (D) Anti-mGlu2/3 receptor staining in 3 week old Nogo-A^-/-^ mice reveals a significant decrease in receptor expression in the hippocampal CA3 area, but not in CA1. Scale bars represent 200 μm. (E) High magnification areas from stratum radiatum in WT and Nogo-A-/- animals. Scale bars represent 10 μm. T-tests were used for all, except D, where Mann-Whitney tests were used.

The lowered theta frequency in Nogo-A^-/-^ hippocampus is similar to the reduction we observed previously in mGlu3^-/-^ mice [26], suggesting a modulation of the mGlu3 receptor by Nogo-A. Evidence for this idea was obtained in experiments in which we tested the effects of pharmacologically blocking the mGlu3 receptor on oscillatory responses in WT and Nogo-A^-/-^ hippocampal slices. In WT hippocampi, as shown previously, application of LY-341495 (3 μM), a group II mGlu receptor antagonist, reduced the frequency of methacholine-induced theta oscillations (from 13.8 ± 1.2 to 9.1 ± 0.4 Hz; *P* = 0.016) as well as the frequency of theta episodes (from 10.6 ± 1.7 to 8.3 ± 1 episodes/min; *P* = 0.304; Fig 5A) in CA3 pyramidal cells through an action mediated by the mGlu3 receptor [26]. Interestingly, this modulation of theta oscillations in response to mGlu3 receptor blockade was absent in the CA3 network of Nogo-A^-/-^ mice (frequency: WT = 9.1 ± 0.4 Hz, Nogo-A^-/-^ = 10.2 ± 0.6 Hz *P =* 0.113; episodes/min: WT = 8.3 ± 1, Nogo-A^-/-^ = 7.5 ± 2.29, *P* = 0.77; Fig 5A). In a second set of experiments we induced hippocampal theta oscillations by selective pharmacological activation of group II mGlu receptors [26]. The frequency of theta oscillations and theta episodes in response to application of LCCG-I (10 μM), a group II mGlu receptor agonist, was reduced significantly in Nogo-A^-/-^ and showed a trend towards reduction in mGlu3^-/-^ hippocampal slices as compared to wild type (frequency: WT = 13.9 ± 0.8 Hz, Nogo-A^-/-^ = 11.4 ± 0.7 Hz, mGlu3^-/-^ = 10.7 ± 0.6 Hz, *P (WT-Nogo-A*^*-/-*^*) =* 0.034, *P (WT-* mGlu3^-/-^*) =* 0.008; episodes/min: WT = 17.2 ± 2.8, Nogo-A^-/-^ = 7.0 ± 2.0, mGlu3^-/-^ = 12.3 ± 2.7; *P (WT-Nogo-A*^*-/-*^*) =* 0.011, *P (WT-* mGlu3^-/-^*) =* 0.241; Fig 5B). Activation of mGlu3 receptors by DCG-IV also induced an inward current in CA3 pyramidal cells from WT mice (at 1 μM: -17.9 ± 5.4 pA, at 3 μM: -29 ± 2.1 pA; Fig 5C) as reported previously [26]. Interestingly, application of DCG-IV failed to induce a significant current in CA3 pyramidal cells in Nogo-A^-/-^ (at 1 μM: 0.1 ± 2.8 pA; *P* = .001 at 3 μM: -1.5 ± 2.6 pA; *P* = .0006) and mGlu3^-/-^ (at 3 μM: -2.2 ± 1.4 pA; *P* = .0001) hippocampus (Fig 5C). Taken together these data are consistent with a downregulation of mGlu3 receptors in CA3 pyramidal cells of Nogo-A^-/-^ mice.

We therefore investigated whether reduction may occur at the RNA or protein level. First, qPCR was performed on RNA from isolated CA3 tissue. No significant reduction of *Grm3* transcript was found in Nogo-A^-/-^ mice (94.6 ± 8.4% of WT; *P* = 0.975; WT: n = 17 mice; Nogo-A^-/-^: n = 7). However, when protein expression was examined with immunohistochemically stained hippocampal slices, we found a significant downregulation of mGlu2/3 receptors in CA3 pyramidal cells, but not in CA1 pyramidal cells of Nogo-A^-/-^ mice (CA3 ROD: WT = 1 ± 0.049, Nogo-A^-/-^ = 0.73 ± 0.038, n = 9; *P* = 0.004; CA1 ROD: WT = 1 ± 0.13, Nogo-A^-/-^ = 0.877 ± 0.11, n = 9; *P* = 0.75. Fig 5D and 5E). No significant reduction was seen in Neun (S3 Fig), used as a control protein. While this reduction likely reflects a loss of the mGlu3 rather than the mGlu2 receptor based on our previous findings [26], there are currently no validated antibodies that definitively distinguish between the two receptors. We therefore designed an experiment to check for a modulation of the mGlu2 receptor by Nogo-A by recording from dentate granule cells, which express functional mGlu2, but not mGlu3 receptors in the somatodendritic compartment [27]. No significant difference in the currents induced by DCG-IV (3 μM) in granule cells was detected between WT, Nogo-A^-/-^, and mGlu3^-/-^ (WT = 47.3 ± 5.7 pA, Nogo-A^-/-^ = 44.6 ± 4.2 pA, mGlu3^-/-^ = 53.6 ± 7.8 pA, *P (WT-Nogo-A*^*-/-*^*) =* 0.746, *P (WT-* mGlu3^-/-^*) =* 0.529), indicating that the expression of mGlu2 receptors is not reduced in granule cells from Nogo-A^-/-^ mice. These data suggest that loss of Nogo-A downregulates mGlu3, but not mGlu2 receptors in CA3 pyramidal cells.

### Absence of Nogo-A increases dependence on global reference frames during spatial navigation in the Morris water maze

Alterations in theta oscillations are frequently associated with deficiencies in spatial navigation [28]. Previous studies, however, have provided conflicting reports on the performance of Nogo-A^-/-^ animals tested in classical Morris water mazes [22,29,30]. Such variance may be attributed to subtle differences in testing environments. As CA3 pyramidal cell function, which is known to be sensitive to spatial cues [31–33], was disrupted in the absence of Nogo-A, we decided to investigate whether search strategies in Nogo-A^-/-^ mice are impaired in a navigational task in which distal visual cues were selectively manipulated.

**Fig 6:**
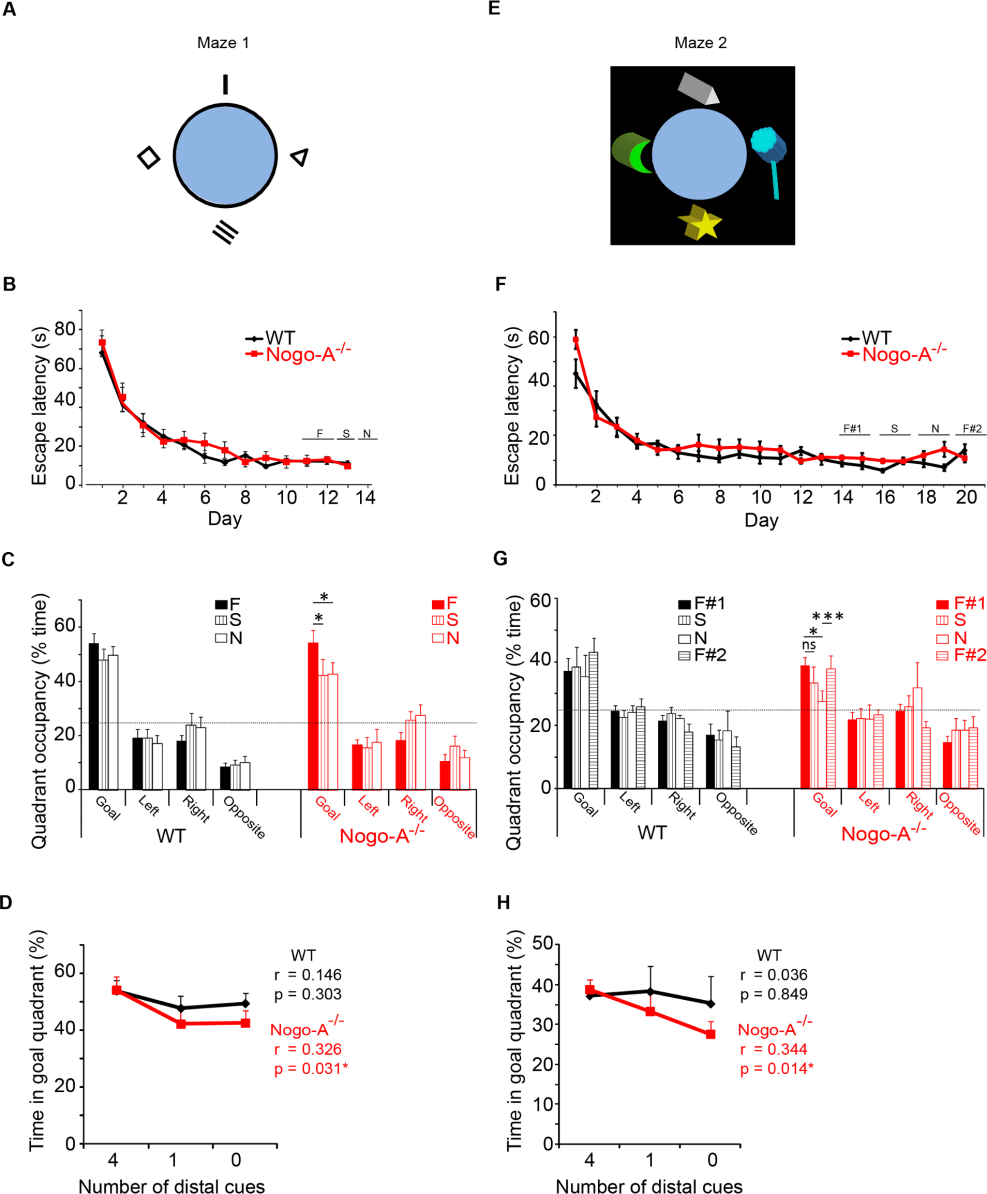
Nogo-A^-/-^ mice exhibit altered spatial navigation strategies. (A) Diagram of Morris water maze 1. (B) Mean escape latency during training trials in maze 1. (C) Probe trial performances in maze 1 (WT: n = 13, Nogo-A^-/-^: n = 11). Probe trial conditions indicated as F (full-cue), S (single-cue) and N (no-cue), t-tests. Full-cue performance is the average of probes on days 11 and 12. The dashed line indicates the level of chance. (D) Pearson’s test reveals a positive correlation between the percent time spent searching the goal quadrant and the number of distal cues for Nogo-A^-/-^, but not WT mice. (E) Diagram of Morris water maze 2. (F) Mean escape latency during training trials in maze 2. (G) Probe trial performance in maze 2 (WT: n = 5, Nogo-A^-/-^: n = 9). Percent time reflects an average of duplicate probe trials. F#1 indicates the first round of full-cue probe trials and F#2 indicates the final round of full-cue probe trials (t-tests). (H) Pearson’s test revealed a significant positive correlation between percent time spent searching the goal quadrant and the number of distal cues for Nogo-A^-/-^, but not WT mice.

Two maze designs were used. Maze 1 was in a well-lit room with four two-dimensional cues on the surrounding walls (Fig 6A). 13 WT mice (19–22 wks) and 11 Nogo-A^-/-^ littermates were subjected to six training trials per day and learned the task at similar rates (Fig 6B, S1 Table and S4 Fig). At the beginning of day 11, mice were subjected to a probe trial with all distal cues in place (full-cue condition) while search behavior was recorded. To ensure a steady level of learning had been achieved, an additional full-cue probe trial was performed on day 12 and data averaged with that of day 11. Both WT and Nogo-A^-/-^ mice spent the majority of their time searching the goal quadrant (WT: 54.1 ± 3.6% in goal, 15.3 ± 1.2% non-goal, *P* = < 0.001; Nogo-A-/-: 54.3 ± 4.5% in goal, 15.2 ± 1.5% non-goal, *P* = < 0.001; S4 Fig), with no significant difference between the two cohorts (WT: 54.1 ± 3.6%, Nogo-A^-/-^: 54.3 ± 4.5%; *P* = 0.96; Fig 6C). The following day, a probe trial was performed with three of the four distal cues removed. Nogo-A^-/-^ mice spent significantly less time searching the goal quadrant than during the full-cue condition (42.4 ± 4.4%, *P* = 0.044), while WT mice were unaffected (47.9 ± 4.3%, *P* = 0.22). A final probe trial was then performed with all four distal cues removed. Again, Nogo-A^-/-^ mice performed significantly worse under this condition than they had with all 4 cues present (42.8 ± 4.4%, *P* = 0.05) and WT mice were unaffected (49.6 ± 3.4%, *P* = 0.26). The lack of decline in Nogo-A^-/-^ performance from single to no-cue was likely due to uncontrolled visual background cues in the room, preventing a significant reduction in distal spatial information by the removal of one additional cue. A Pearson correlation test of all probe trials from Nogo-A^-/-^ mice showed a positive correlation between the number of distal cues present and percent time searching the goal quadrant (r(42) = 0.326, *P* = 0.031). No correlation was detected in WT animals (r(50) = 0.146, *P* = 0.303; Fig 6D).

Maze 2 was designed to be more challenging, in that lighting was decreased to limit local reference frames (i.e. pool walls) (Fig 6E). The pool was surrounded by black curtains that provided a dark environment with four illuminated three-dimensional cues (see methods and materials). Naïve mice were trained as those in maze 1, with WT and Nogo-A^-/-^ animals learning the task at a similar rate (Fig 6F, S1 Table, and S4 Fig). On days 14 and 15, full-cue probe trials were conducted and performances were averaged. Both WT and Nogo-A^-/-^ mice trained in maze 2 spent significantly less time searching the goal quadrant than those in maze 1 (WT: maze 1: 54.1 ± 3.6% n = 13; maze 2: 31.3 ± 2.8% n = 10, *P* < 0.001; Nogo-A^-/-^: maze 1: 54.3 ± 4.5% n = 11; maze 2: 34.2 ± 2.7% n = 13, *P* < 0.001). Only animals that showed preference for the goal quadrant (frequented more than any other quadrant during the full-cue probe trial) were used for the remaining experiments (WT: 37.2 ± 4% in goal, 20.9 ± 1.3% non-goal, n = 5, *P* = .037; Nogo-A-/-: 38.9 ± 2.4% in goal, 20.4 ± 0.8% non-goal, n = 9, *P* = < 0.001; S4 Fig). Of these mice, there was no difference in performance between WT and KO animals (WT: 37.2 ± 4%, Nogo-A^-/-^: 38.9 ± 2.4%, *P* = 0.71; Fig 6G). Single-cue probe tests revealed no decline in WT performance (38.4 ± 6.3%, *P* = 0.84), but a slight decrease within the Nogo-A^-/-^ group (33.4 ± 5.1%, *P* = 0.17). Removal of all four distal cues caused a significant reduction in performance of Nogo-A^-/-^ animals (27.7 ± 3.3%, *P* = 0.024), while the WT group was unaffected (35.3 ± 6.8%, *P* = 0.69). We further compared WT and KO groups based on the performance during both mazes combined. An ANOVA test revealed that among mice capable of learning the task when all distal cues were present, the Nogo-A^-/-^ group performed significantly worse than did the WT group in the no distal-cue condition (n = 18 for WT and 20 for KO, [F(1,36) = 4.23, *P* = 0.047]). It is possible that the poor spatial navigation shown by Nogo-A^-/-^ mice during the no-distal cue probe trial was caused by memory extinction through exposure to the single-cue environment. To test this hypothesis, we performed probe trials with all four cues restored. Nogo-A^-/-^ mice showed significant improvements under this condition (37.9 ± 4.1%, *P* = 0.001) and had therefore not lost their ability to use distal cue-based navigation. Performance in WT mice increased modestly and did not approach significance (43 ± 4.6%, *P* = 0.25). A Pearson correlation test of all probe trials revealed a significant relationship between performance and the number of distal cues for Nogo-A^-/-^ (r(52) = 0.334, *P* = 0.014), but not WT mice (r(28) = 0.036, *P* = 0.849; Fig 6*H*). Furthermore, a one-way ANOVA revealed a highly significant difference among cue conditions on Nogo-A^-/-^ animals when examining all probe trials from all animals used in both mazes (F(2,95) = 7.173, *P* = < 0.001). A Tukey post hoc test test revealed significant differences between full-cue and single-cue conditions (*P* = 0.023), as well as between full-cue and no distal-cue conditions (*P* = 0.002). In contrast, no significant effect was seen in WT mice (F(2,79) = 1.157, *P* = 0.320).

## Discussion

We have shown that Nogo-A mediates critical functions of CA3 pyramidal cells that are important in the generation of hippocampal network responses. The data indicate that Nogo-A modulates CA3 excitation/inhibition ratios, network oscillatory, and regulates the expression of mGlu3 metabotropic glutamate receptors in the postsynaptic compartment of pyramidal cells, which may account for the increased dependence on distal reference frames during spatial navigation.

### Alteration of CA3 network responses in Nogo-A^-/-^ mice

Examination of spontaneous activity in the CA3 area revealed that the frequency of EPSCs, but not of IPSCs was increased in CA3 pyramidal cells in the absence of Nogo-A^-/-^. This is in contrast with the CA1 network, where loss of Nogo-A caused no changes in spontaneous activity. Within CA3, no change in the synaptic responses of interneurons was observed, in agreement with HPLC determinations showing no changes in GABA concentration in these mice [15]. The increase in excitatory synaptic responses may reflect the greater dendritic complexity in pyramidal cells in the absence of Nogo-A [4] and Fig 1*A*. The increase in spontaneous EPSC frequency was associated with a hyperactive state of the CA3 neuronal network, marked by periods of high frequency activity, which may be accounted for by a more predominant role of Nogo-A in pyramidal cells than in inhibitory interneurons (Fig 2). However, these bouts of aberrant responses did not engender epileptiform discharge, which is consistent with the behavioral phenotype of Nogo-A^-/-^ mice where seizures have not been observed [29,34,35]. The alterations in dendritic arborization reported for Nogo-A^-/-^ mice were observed primarily in regions where recurrent collateral synapses among CA3 pyramidal cells occur, as reported previously [4].

We noted disruptions of theta rhythmicity in hippocampus lacking Nogo-A, consistent with the critical role of CA3 recurrent collateral synapses in the generation of oscillatory activity patterns [24,36]. It has been shown that the frequency of theta episodes can significantly impact performance of navigational tasks in rats [28]. It is therefore plausible that the observed decrease in theta occurrence in Nogo-A^-/-^ CA3 networks contributes to altered spatial navigation strategy. Oscillation frequency within theta episodes was also decreased in KO mice. This finding supports previous proposals that Nogo-A is an influential protein in schizophrenia, as patients have been reported to exhibit reduced theta oscillation frequency during certain tasks [37]. And while our examination of oscillatory activity was carried out *in vitro*, previous studies have shown a strong correspondence between *in vitro* and *in vivo* hippocampal theta episodes [38,39].

Several lines of evidence support a common mechanism that may underlie our findings whereby postsynaptic mGlu3 receptors in CA3 pyramidal cells are downregulated in the absence of Nogo-A.

The alterations in theta oscillations are similar to those we observed from the hippocampus of mGlu3^-/-^ mice [26], and the difference between WT and Nogo-A^-/-^ theta is no longer observed in the presence of an mGlu2/3 antagonist. Furthermore, in both Nogo-A^-/-^ as well as in mGlu3^-/-^ CA3 pyramidal cells, the application of an mGlu2/3 agonist failed to induce inward currents. Finally, immunohistochemistry revealed that the expression of mGlu3 receptors in the CA3 area is significantly reduced in the absence of Nogo-A. Although antibodies against mGlu3 also label mGlu2, our finding that selective mGlu2 receptor-mediated responses were unchanged in neighboring Nogo-A^-/-^ dentate granule cells, which display high expression of this receptor [27], further suggests a selective action of Nogo-A on mGlu3 receptors.

There are multiple mechanisms through which Nogo-A may regulate mGlu3 receptors. mGlu3 expression is known to be particularly sensitive to neuronal activity [40]. Thus, hyperexcitability of the Nogo-A^-/-^ CA3 network may induce activity-dependent downregulation of the receptor. Alternatively, Nogo-A may play a role in trafficking mGlu3 to the membrane. And while qPCR results suggest a lack of transcriptional regulation, translation may be impaired in Nogo-A^-/-^ cells. Our future work will be directed at characterizing the molecular mechanisms underlying the relationship between these two proteins.

As the mice used in this study had global deletion of Nogo-A, we cannot rule-out possible effects from altered glia cells. Indeed, a recently study by Zemmar et al.[41] showed that both neuronal and oligodendrocyte-localized Nogo-A regulate dendritic branching in cortical neurons. Interestingly, the two had compartmentalized effects, with oligodendrocytic Nogo-A regulating distal dendrites and neuronal Nogo-A modulating more proximal dendrites. We observed effects on dendritic arborizations only in relatively proximal areas (*stratum radiatum*) of CA3 pyramidal cells, where recurrent collaterals contribute most to theta oscillations. However, it cannot be excluded that loss of oligodendritic Nogo-A impacts synaptic activity in this region. While astrocytic alterations, such as potassium buffering and glutamate uptake, could certainly affect neuronal activity, these cells are known to express very little Nogo-A[42].

It has been shown that Nogo-A^-/-^ animals display a mild (10%) increase in levels of Nogo-B[43], which could contribute to the observed changes in synaptic activity. However, this upregulation occurred mostly in oligodendrocytes. And while Nogo-A is present in the synapse, neuronal Nogo-B is believed to exist predominantly in extrasynaptic regions[44]. Therefore, it is most likely that the changes in neuronal activity we observed are due to a loss of Nogo-A and not an increase in Nogo-B.

### Effects on spatial navigation

Although it was long believed that rodents utilize distal cues, that is, global reference frames, as the only source of orientation in the MWM, accumulating evidence suggests otherwise. Recent work has demonstrated the importance of local reference frames, particularly apparatus boundaries, seemingly non-polarized, in the localization of target platforms (for review see [45]). It is well established that CA3 place cells have a tendency to map according to local cues, whereas those in CA1 show a preference for distal cues [31,33,46]. We thus predicted that Nogo-A^-/-^ mice, with disrupted CA3 circuitry, would rely less on local reference frames and more heavily on distal cues. Indeed, when distal cues were removed, Nogo-A^-/-^ mice exhibited significant impairment in probe trials.

Our finding that wild-type mice are capable of navigating with reduced distal information could imply that extraneous auditory or olfactory cues were not controlled for. However, mice tested in maze 2, where only visual aspects of the environment were altered (i.e., decreased saliency of the pool wall using low light), performed significantly worse than did mice in maze 1 during the full-cue condition. This suggests that mice were relying primarily on visual cues, although additional use of non-visual information cannot be fully excluded.

**Fig 7:**
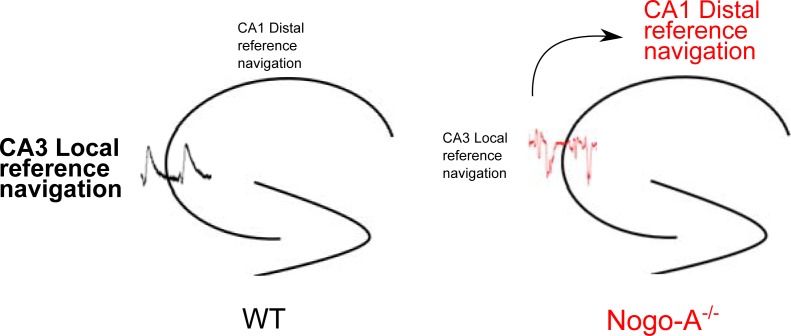
Proposed effects of hippocampal Nogo-A loss. Down-regulation of Nogo-A specifically disrupts CA3 network activity and theta rhythmicity. As a result, spatial navigation strategies are shifted from a predominantly CA3-regulated behavior (local reference frame processing) to a CA1-regulated behavior (distal reference frame processing).

The ability of mutant mice to process distal cues, involving primarily CA1 cells, was not unexpected, as previous studies have shown Nogo-A to be weakly expressed in the CA1 area and that the constitutive absence of Nogo-A does not significantly modify synaptic properties at Schaffer collateral inputs onto CA1 pyramidal cells [3,4]. Furthermore, we observed no alterations to spontaneous activity in the CA1 region of Nogo-A^-/-^ mice. On the basis of these findings we propose that the disruption of CA3 network function impairs processing of local reference frames (i.e. apparatus boundaries) and shifts dependence to distal reference frame-based navigation (Fig 7). Future studies using *in vivo* recordings will be needed to confirm this hypothesis.

In conclusion, we have shown that Nogo-A^-/-^ mice exhibit increased hippocampal activity, disrupted rhythmicity attributed to downregulation of mGlu3 receptors and altered spatial navigation. These findings are of particular interest, as hippocampal hyperexcitability, altered network oscillations, and impaired navigation have been described not only in other schizophrenia animal models [47,48], but also in patients with schizophrenia [49,50]. Previous studies have drawn correlations between both Nogo-A and mGlu3 receptor alterations to psychiatric illnesses. Indeed, single nucleotide polymorphisms located in *Grm3* (mGlu3) or *Rtn4* (Nogo-A) genes are positively associated with schizophrenia [11,12,51,52] and deletion of either is known to induce related endophenotypes in mice [15,53]. While there is a long list of candidate genes for psychiatric diseases, which typically display multifactorial etiologies, in most cases potential interactions have not been determined. The characterization of a functional link between Nogo-A and mGlu3 receptors, may thus provide novel insights into the underlying pathophysiology of schizophrenia.

## Supporting information

S1 Table Detailed results of statistical tests. (XLSX)

S1 Fig The laura antibody binds specifically to Nogo-A. Representative images of Nogo-A staining in wildtype and knockout animals. Immunoflourescense is not detected in Nogo-A^-/-^ tissue. Scale bars represent 300 μm.
(EPS)

S2 Fig Anti-mGlu2/3 specificity. Immunoflourescence is significantly attenuated when pre-incubating the mGlu2/3 antibody with a synthetic mGlu2/3 peptide fragment. Scale bars represent 10 μm, Mann-Whitney test.
(EPS)

S3 Fig NeuN staining is not down-regulated in Nogo-A^-/-^ slices. Representative images of NeuN staining in wildtype and Nogo-A^-/-^ tissue. Scale bars indicate 100 μm.
(EPS)

S4 Fig Wildtype and Nogo-A^-/-^ mice learn the basic MWM task equally well. (A) Maze 1.Left, mice learn the task at a similar rate, as judged by performance in the first trial of each day. Right, both cohorts spend significantly more time searching the goal quadrant than other quadrants during the full-cue probe trials (t-tests) (B) Same as A, but for maze 2.
(EPS)
